# Melanoma development: stage-dependent cancer competence of the melanocytic lineage

**DOI:** 10.1038/s41392-021-00854-3

**Published:** 2021-12-20

**Authors:** Manfred Kunz

**Affiliations:** Department of Dermatology, Venereology and Allergology, University Medical Center Leipzig, Philipp-Rosenthal-Str. 23, 04155 Leipzig, Germany

**Keywords:** Skin cancer, Cancer genetics

In a recent article in *Science*, Baggiolini et al.^1^ showed that the transforming ability of the *BRAF*V600E oncogene for melanoma development depends on the stage of the transformed melanocyte lineage cells. While neural crest cells and melanoblasts are vulnerable to malignant transformation in a zebrafish model, melanocytes only developed small patches of nevus-like cells after *BRAF*V600E expression.

**Fig. 1:**
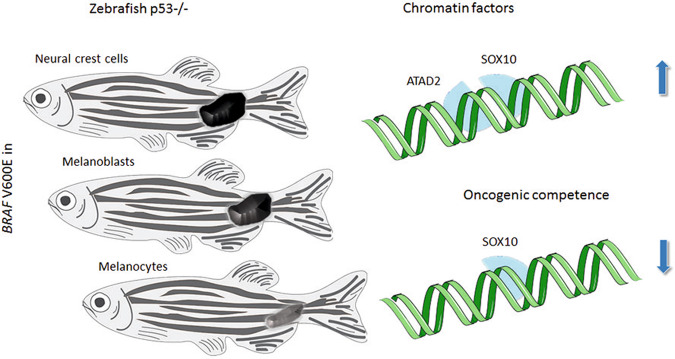
Oncogenic transformation of p53−/− zebrafish results in melanoma formation when the melanoma oncogene *BRAF*V600E is expressed in neural crest cells or melanoblasts but not when expressed in melanocytes. Epigenetic analyses showed that ATAD2–SOX10 interaction plays a key role in this process

The tumorigenic potential of DNA mutations depends on the transcriptional and developmental stage and the cellular context in a number of different cancers.^2^ However, this phenomenon has not been studied in detail in malignant melanoma, one of the most aggressive cancers with high metastatic potential. In the mentioned study by Baggiolini et al.,^1^ a set of elegant molecular experiments in zebrafish and mice was performed together with additional in vitro experiments. Melanocyte precursors such as neural crest cells and melanoblasts produced aggressive melanomas in p53−/− zebrafish, engineered to express the melanoma oncogene *BRAF*V600E, while differentiated melanocytes did not (Fig. 1). In these experiments, expression of the *BRAF*V600E oncogene was put under the control of stage-specific promoters of melanocyte lineage cells, i.e., *sox10* for neural crest cells, *mitf* for melanoblasts and *tyrp1* for melanocytes.^1^ To further corroborate these findings, human pluripotent stem cells (hPSC) were differentiated into neural crest cells, melanoblasts and mature melanocytes on a triple gene knockout background (3xKO cells). 3xKO stands for a knockout of tumor suppressor genes *RB1*, *TP53*, and *p16. BRAF*V600E expression was put under the control of a doxycycline-dependent promoter. Subcutaneous injections of either transformed neural crest cells or melanoblasts resulted in tumor formation in immunodeficient NOD scid gamma mice, while injections of transformed melanocytes did not. As shown by RNA-seq analysis, it was further shown that neural crest cells and melanoblasts clustered together with expression profiles of melanoma patient samples of The Cancer Genome Atlas (TCGA). Gene set enrichment analysis showed that pathways enriched in melanoblasts compared to melanocytes were related to chromatin modification, suggesting that epigenetic factors with impact on the chromatin state made these cells competent for melanoma development. Among the top epigenetic-related factors was *ATAD2* (ATPAse family AAA domain containing 2). By lentivirus-based induction of *ATAD2* expression in 3xKO melanocytes, to establish a progenitor signature, it was subsequently shown by transposase-accessible chromatin analyses (ATAC-seq), that ATAD2 expression opened chromatin at neural-crest related loci. Among transcription factors active at these loci, SOX10 was identified as top candidate. Thus, ATAD2 may specifically support SOX10 in its binding to target genes. Gene network analysis showed an enrichment of pathways associated with neural crest proliferation and migration. Inactivation of *ATAD2* reduced the percentage of neural-crest cell formation in hPSC cells. In line with this, it is well-understood that SOX10 expression is required for melanoma formation in *NRAS*-mutant mouse melanomas.

TCGA patients with an ATAD2-high signature also showed an enrichment of the SOX10 motif, as well as a strong Myc signature.^1^ In co-immunoprecipitation experiments ATAD2 formed protein complexes with SOX10 and Myc. Co-binding of ATAD2 and SOX10 to ATAD2 target genes was further demonstrated and resulted in the enrichment of the expression of neural-crest related genes. Moreover, genes of the mitogen-activated protein kinase (MAPK) pathway were upregulated in these experiments with 3xKO cells, including epidermal growth factor receptor (EGFR) and fibroblast growth factor receptor 2 (FGF2), which may have an impact of ERK1/2 phosphorylation. This indicates that ATAD2 impacts on neural crest lineage programs and MAPK pathway activation. In a next set of experiments, 3xKO *ATAD2* melanocytes were more invasive in invasion chamber assays than 3xKO cells without *ATAD2* induction.

In a final set of experiments, recapitulating the initial experiments, a transgenic zebrafish was generated in which *BRAF*V600E was overexpressed together with *ATAD2* in a p53−/− background to test whether *ATAD2* expression might be sufficient for melanoma development in a setting using the *tyrp1* promoter. While tyrp1-dependent *ATAD2* expressing cells developed melanomas in 10%, with an additional 15% who developed hyperplastic lesions, none of the *ATAD2* non-induced cells developed melanomas. After electroporation of the zebrafish with single-guide RNA targeted against *ATAD2*, a significant decrease in tumor size was observed. Together, high ATAD2 levels supported the re-expression of a progenitor signature and may thus help mutant *BRAF* to initiate melanomas.

These findings present in a conclusive way strong experimental evidence that oncogenic pathways must be active at a vulnerable stage of cell development to induce tumor formation, which obviously depends on the cellular context. In a recent study by Belote et al.,^3^ transcriptional patterns of melanocytes from different stages of development were analyzed to provide an atlas of human epidermal melanocytes. It was shown that neonatal melanocyte signatures were associated with high treatment resistance and low overall survival in melanoma patients.^3^ Surprisingly, melanocyte stem cell and fetal melanocyte signatures were associated with a better prognosis, but still worse than mature melanocyte signatures. However, survival was largely based on data from treatment with immune-modulatory substances, which were not analyzed in the present study. Moreover, an earlier report on mouse melanocytes showed that melanoblast transcriptomes were indeed associated with melanoma metastasis.^4^ For the moment, Baggiolini et al.^1^ cannot completely explain why established mouse melanoma models where a tyrosinase Cre driver that activates *BRAF*V600E induces melanomas in the context of *PTEN*, *CDKN2A*, and *TP53* inactivation.^5^ They argued that the melanoma cells of origin in these experiments have not fully been identified and that the *tyrp1* promoter used in the present study expressed the transgene in a more differentiated type of cells than the tyrosinase (trp) promoter. It is well understood that malignant transformation of melanocytes in vitro has been difficult to obtain with a limited number of oncogenic variants, which supports the notion that differentiated melanocytes might indeed not be the common precursor cells for melanoma. Collectively, the present paper points into a new direction in the understanding of melanoma biology based on an epigenetic re-programming in early stages of melanocyte precursors, which helps to better understand why melanocytes rarely transform into tumors.
